# Topical Antioxidant Cocoa Polyphenol Skin Penetration

**DOI:** 10.1111/jocd.16637

**Published:** 2024-10-21

**Authors:** Zoe Diana Draelos, Matthew M. Draelos, Sue Feng, Zydnia Madera, Mia Chen

**Affiliations:** ^1^ Dermatology Consulting Services, PLLC High Point North Carolina USA; ^2^ ET Browne Englewood Cliffs New Jersey USA

**Keywords:** catechin, cocoa polyphenols, epicatechin

## Abstract

**Background:**

Cocoa powder derived from the *Theobroma cacao* plant is rich in polyphenols, such as catechin and epicatechin. These polyphenols are strong antioxidants when consumed orally; however, their ability to enter the stratum corneum following the topical application has never been demonstrated.

**Objective:**

The objective of this study was to demonstrate the deposition of catechin and epicatechin in the stratum corneum following the topical application of 6% aqueous cocoa powder 1 and 2 h after application.

**Methods:**

Five healthy female subjects aged 25–60 years were enrolled. A 6% aqueous cocoa powder solution was prepared and applied to two randomized designated spots on the left forearm. 2 cc of the solution was applied under a ¼‐inch gauze square covered with plastic wrap and held in place with a Coban dressing. The 15 d‐squame 7/8‐inch circular tape strips were applied to the predetermined area on the forearm 1 h and 2 h after application of the 6% cocoa powder solution. The tape strips were immediately placed in a −80°C freezer for storage until extraction in preparation for LC–MS evaluation for catechin and epicatechin levels.

**Results:**

More catechin and epicatechin were detected at 2 h than 1 h for both compounds, although the difference was not statistically significant. Higher epicatechin levels than catechin levels were detected from the cocoa powder at both time points. This is consistent with published data from food‐grade cocoa powder.

**Summary:**

It is detected that 6% aqueous cocoa powder delivers the antioxidants catechin and epicatechin to the stratum corneum 1 h and 2 h after topical application.

## 1 Introduction

The earliest human use of cocoa has been dated to 2000 BC based on the dating of residue on drinking cups and plates discovered in Latin America in a small village in Honduras. Cocoa, derived from the *Theobroma cacao* plant, was also a key component of Mayan agriculture in 900 AD and was brought to Europe by the Spanish in 1505. It was used for medicinal purposes to act as a stimulant and was found in many 17th‐ and 18th‐century medications [1]. Cocoa liquor is prepared from the dried and fully fermented seed of the fruit of the cocoa tree, known as the cocoa bean, that is made into a paste. Removing some of the cocoa butter from the liquor makes cocoa powder.

Cocoa powder is very rich in methylxanthines, such as caffeine, theobromine, and theophylline. It is a polyphenol‐rich food containing a variety of flavonols, including catechin and eipcatechin, which possess potent antioxidant properties via free radical trapping and chelation of redox‐active metals [2]. Cocoa powder contains up to 50 mg of polyphenols per gram [3]. These rich polyphenols are not found in cocoa butter [3]. Prior methods of determining the availability of the polyphenols examined their concentration in urine and plasma after ingestion of cocoa powder.

Cocoa can be topically applied to the skin to provide protection from oxidative damage induced by UV radiation [4]. This may be due to down regulation of pro‐inflammatory cytokines and their downstream biochemical pathways [5]. For example, cocoa has been shown to inhibit NF‐κB activation, resulting in a reduction in IL‐2 production [6]. Cocoa powder and cocoa butter are used in topical creams and lotions for their ability to soothe irritated, inflamed skin, but no studies have been conducted to demonstrate the ability of cocoa to enter the stratum corneum and exert these skin benefits. This study was designed to assess the ability of 6% cocoa powder to deposit in the stratum corneum using tape stripping and liquid chromatography‐mass spectrometry (LC–MS). Catechin and epicatechin were selected as target analytes for identification. Our study demonstrates and quantitates the amount of deposited aqueously extracted cocoa powder‐derived catechin and epicatechin in the stratum corneum at a given depth over time.

## 2 Methods

### 2.1 Clinical Design

Five female subjects aged 25–60 years with no known medical conditions on their forearms who signed informed consent (Allendale Institutional Review Board [AIRB], Old Lyme, CT) were enrolled. A 6% aqueous cocoa powder solution was prepared and applied to two randomized designated spots on the left forearm. A volume of 2 cc of the solution was applied under a ¼‐inch gauze square covered with plastic wrap and held in place with a Coban dressing. The subjects sat quietly in a temperature (70°F–74°F) and humidity (30%–40%) controlled environment. The Coban and gauze were removed 50 min after application for the 1 h solution contact site and 110 min after application for the 2 h solution contact site.

The 15 d‐squame 7/8‐inch circular tape strips (Clinical & Derm, LLC) were applied to the predetermined area on the forearm 1 h and 2 h after application of the aqueous 6% cocoa powder solution. The plunger was held in place for 10 s exerting a controlled 225 g per centimeter squared pressure to ensure adequate and consistent contact between the tape and the skin. The first tape strip removed was discarded to remove any excess cocoa powder solution sitting on the skin surface. This procedure was repeated 15 times on the same area with a new tape strip, and each tape strip was removed with forceps and placed in a separate clean specimen jar. The technician took care not to handle or contaminate the tape strips during the application or removal process. Care was also taken to exactly overlap the sampling location of the tape strips. Fifteen tape strips were also taken from a control subject who did not have any contact with the cocoa powder solution. The tape strips were immediately placed in a −80°C freezer for storage until they could be solvent extracted in preparation for LC–MS quantitation of catechin and epicatechin levels. This process generated 90 samples, which was felt to be an adequate sample size for analysis to identify the presence and amount of the cocoa polyphenols epicatechin and catechin in the skin.

### 2.2 Internal Standard and Standard Curve Preparation

The (−)‐epicatechin and (+)‐catechin analytical standards were purchased from commercial sources and used without further purification (MilliporeSigma). Epicatechin and catechin external standards and a tazarotene internal standard were prepared with methanol solvent using standard analytical scales, calibrated pipettes, and class A volumetric flasks. A tazarotene internal standard solution was prepared with 2.39 mg weighed onto an analytical scale and diluted with 1 L of methanol prior to analysis and stored in a sealed glassware at −80°C when not in use. 2.70 mg of analytical‐grade epicatechin and 3.94 mg of analytical‐grade catechin were weighed on a standard analytical scale and serially diluted in methanol to generate standard curves for these compounds. The standard curve was generated in triplicate.

### 2.3 Sample Preparation and Analysis

The 20‐mL sample vials containing the tape strips were removed from the freezer and allowed to warm to room temperature. Then, 2 mL of methanol spiked with the tazarotene internal standard was added. The vials were sonicated for 2 min at 25°C followed by manual inversion to ensure consistent mixing. Next, 1 mL of the extraction solution was filtered with a 0.2 μm nylon filter into a 1.5 mL vial with an unperforated PTFE septum suitable for high pressure liquid chromatography (HPLC) assessment. The samples were placed in the HPLC auto‐sampler carousel and cooled to 4°C during analysis.

Initially, each sample was analyzed with an injection of 1 μL into the LC–MS instrumentation. Although excellent peak shape was obtained, the signal was at the limit of quantitation for tape strips 1 through 5 and below the limit of detection for tape strips 7 and 9. Therefore, each sample was re‐injected with an injection volume of 5 μL. A single sample, C‐1‐1‐2, was re‐injected with a volume of 7 μL. Due to the re‐injection, the samples were stored on the auto‐sampler carousel at 4°C for approximately 24 h. Quality control injections after approximately 24 h demonstrated no loss of signal for epicatechin or catechin in the external standards, providing confidence in the below findings, despite the extended duration of the analysis.

### 2.4 Analytical Equipment and Method

LC‐ESI‐MS data was collected on an UltiMate 3000 system consisting of an LPG‐3400SD pump, TCC‐3000SD column oven, WPS‐3000TSL auto‐sampler, and DAD‐3000 diode array equipped with a Hypersil GOLD C18 4.6 × 150 mm^2^ column (Thermo Fisher Scientific) attached to an ISQ EM Single Quadrupole mass spectrometer with atmospheric pressure chemical ionization (Thermo Fisher Scientific; Waltham, MA). Briefly, the HPLC was operated with a flow rate of 1 mL/min with a binary system consisting of H_2_O + 0.1% formic acid (A) and methanol + 0.1% formic acid (B) and an oven temperature of 30°C. Following a 5 minute (min) pre‐equilibration with 65% A; 35% B, the following gradient use: 65% A, 35% B (0–1 min); 65% A, 35% B to 5% A, 95% B (1–3 min); 5% A; 95% B (3–6.9 min). Epicatechin and catechin elution was monitored by ESI‐MS: calculated [M + H]^+^ for C_15_H_15_O_6_ = 291.09 m/z. Tazarotene internal standard elution was monitored by ESI‐MS: calculated [M + H]^+^ for C_21_H_22_NO_2_S = 352.14 m/z. The ESI‐MS was configured with a vaporizer temperature: 550°C; ion transfer tube temperature: 350°C; source voltage: 3000 V; source CID voltage: 5 V; sheath gas pressure: 80 psig; auxiliary gas pressure: 11.7 psig; and sweep gas pressure: 0.0 psig. Data analysis was completed using Chromeleon 7.2 (Thermo Fisher Scientific).

## 3 Results

The catechin and epicatechin underwent extraction from the 6% cocoa powder in an aqueous solution at 1 and 2 h. Our analytical method performed well, with the standard curves for both epicatechin and catechin having *R* ^2^ values of 0.9998 and 0.9998 within the relevant range, respectively. More catechin and epicatechin were detected at 2 h than 1 h for both compounds. Although the difference was not statistically significant, the trend over multiple tape strips suggests that this difference may be statistically significant with more replicates. Higher epicatechin levels than catechin levels were detected from the cocoa powder at both time points. This is consistent with published data from distillate derived from methanol extraction of food‐grade cocoa powder [7]. Compared to these harsh conditions, unsurprisingly less epicatachin and catechin were detected under our gentler analytical conditions. The 6% cocoa powder suspension for cutaneous application was prepared with water, not methanol, and was not boiled prior to application.

**FIGURE 1:**
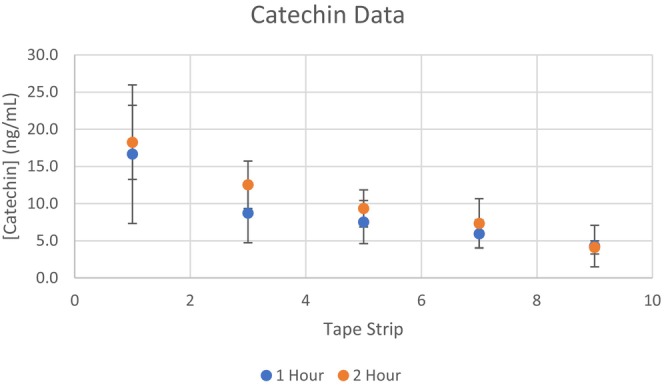
Catechin stratum corneum levels. Catechin stratum corneum levels were low, but detectable 1 h and 2 h after topical application of 6% aqueous cocoa powder.

**FIGURE 2:**
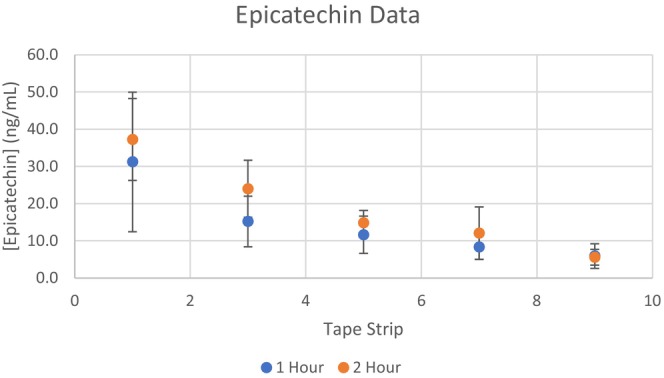
Epicatechin stratum corneum levels. Epicatechin stratum corneum levels were higher than catechin levels, which is consistent with the amounts found in cocoa powder.

Excellent peak shape was obtained at 1 μL injections onto the LC–MS instrumentation, although insufficient signal for epicatechin and catechin was detected for reliable quantitation. Therefore, re‐injection with 5 μL onto the LC–MS instrumentation was necessary to detect the catechin and epicatechin at the expense of broader peaks. Injection volumes greater than 5 μL significantly compromised peak shape for analytical quantitation. The retention time of the quantitated epicatechin and catechin signal had excellent agreement with the retention times of the analytical standards using an injection volume of 1 and 5 μL. Selected samples were also analyzed with a Vanquish Horizon attached to an Orbitrap Exploris 240 with internal calibration for high‐resolution accurate mass (Thermo Scientific) to confirm the detection of epicatechin and catechin with the extracted tape trip solution (data not shown). Furthermore, the relative amounts of catechin and epicatechin detected are in general agreement with the literature [7]. Thus, the presented data likely accurately reflects the amount of epicatechin and catechin deposited within the stratum corneum, despite the complex chemical milieu resulting from the aqueous extraction of cocoa powder. Excellent levels of epicatechin and catechin were seen in tape strips 1 and 3 with sequentially decreasing levels in tape strips 5, 7, and 9 (Figures 1 and 2). Even though the catechin and epicatechin were detected in tape strips 7 and 9, the amounts approached the lower limit of detection and quantitation of our method and equipment.

## 4 Discussion

Cocoa powder contains approximately 13.5% phenols that are present as monomeric epicatechin and catechin, dimeric procyanidins, oligomers, and polymers. More minor polyphenols found in cocoa powder include luteolin, apigenin, narigenin, quercetin, isoquercitrin, and anthocyanins [8]. Cocoa powder polyphenols are approximately 37% catechins, 58% proanthocyanidins, and 4% anthocyanidins [8]. It is for this reason catechin and epicatechin were selected as the two primary antioxidant analytes for LC–MS detection following stratum corneum tape stripping.

Currently marketed skin care products contain numerous botanical ingredients designed to function as antioxidants and anti‐inflammatory agents. However, oxidative damage and inflammation do not occur on the skin surface, but rather within the skin itself. Antioxidants must be deposited into the stratum corneum or further into the skin to have a protective effect. While it is assumed that topical botanicals in skin care formulations when applied topically to the skin have an effect, this must be demonstrated. Many antioxidants become rapidly oxidized when applied to the skin surface and remain active for a very short period of time. Other antioxidants are placed in cosmetic formulations to act as preservatives for the product. For example, vitamins E and A are common antioxidants used in skin care creams and lotions to prevent rancidity or oxidation of the lipids in the formulation. They are present in low concentrations, appearing toward the end of the ingredient disclosure list, possibly possessing insufficient amounts to make much difference when topically applied to the skin. Other antioxidants are lipid soluble and remain bound to an ingredient in the skin‐care vehicle or when they disassociate are able to penetrate less readily than water‐soluble antioxidants due to their lipophilic properties. This research demonstrated a method for identifying the ability of botanical ingredients to function effectively in topical formulations. It is a step forward in validating cosmeceutical formulations in terms of their functionality beyond the ingredient disclosure.

Cocoa powder is water soluble containing stable polyphenols with excellent antioxidant capabilities [9]. Oral cocoa has been shown to provide a variety of skin benefits including enhanced photoprotection [10, 11] and inflammation reduction [12, 13, 14]. Topical cocoa powder in combination with cocoa butter has been used for many years in cosmetic moisturizer formulations to reduce inflammation and enhance the skin barrier. Cocoa butter can function as an occlusive agent placing a lipid‐rich layer over the skin to decrease transepidermal water loss (TEWL) and create an optimal environment for barrier repair, in addition to supplying skin antioxidants. However, cocoa butter does not contain the antioxidants. The addition of cocoa powder provides antioxidants topically, as shown in this research. After 1 h and 2 h of cocoa powder contact with the skin, catechin and epicatechin levels were detectable indicating that the antioxidant was found intact in the stratum corneum down to layer 9. While it might be of interest to assess the amount of cocoa powder polyphenols deeper in the skin, this was not possible as punctate bleeding occurred at tape strip 9 indicating that the viable epidermis had been reached. Once bleeding occurs, the tape strips will no longer stick and this noninvasive method for identifying antioxidant penetration into the skin is no longer valid.

A limitation of this study is the analysis of five subjects plus one control. Fifteen samples were generated per subject for a total of 90 specimens. While there was excellent consistency in the detection of cocoa polyphenols in the skin, additional samples could have provided better confirmation. An additional limitation of this study is that raw cocoa powder contains many thousands of unique compounds, some of which may have an identical retention time and mass as epicatechin and catechin. The possibility of contaminants from cocoa powder affecting the quantitation of epicatechin and catechin cannot entirely be ruled out despite the controls conducted in this research. Data suggesting an accurate quantitation of catechin and epicatechin are (1) the reported ratio of catechin and epicatechin is reasonable and well aligned with the prior literature and (2) there is good agreement between the retention times of the known analytical standards and the signal obtained from the raw cocoa powder tape strips. Regardless, the present data are suggestive of detectable amounts of epicatechin and catechin entering the stratum corneum from topically applied 6% cocoa powder, which may provide antioxidant benefits to the skin.

## 5 Summary

The polyphenol antioxidants/anti‐inflammatories catechin and epicatechin were detected in the stratum corneum 1 and 2 h after application of 6% cocoa powder.

## Author Contributions

Z.D.D. and M.M.D. performed the research; S.F., Z.D.D., M.M.D. designed the research study; Z.D.D., M.M.D., S.F., Z.M., M.C. analyzed the data; Z.D.D. and M.M.D. wrote the paper; all authors read and approved the final manuscript.

## Ethics Statement

The study documents and consent were approved by the Allendale Institutional Review Board (AIRB), Old Lyme, CT.

## Conflicts of Interest

Zoe Diana Draelos, MD, is a researcher for ET Browne. Sue Feng, PhD, Zydnia Madera, and Mia Chen are employees of ET Browne. Matthew M. Draelos, MD, PhD declares no conflicts of interest.

## Data Availability

The data that support the findings of this study are available on request from the corresponding author. The data are not publicly available due to privacy or ethical restrictions.
